# A point-based laminitis risk scoring system and machine learning framework for prediction of laminitis in horses

**DOI:** 10.3389/fvets.2026.1809041

**Published:** 2026-07-01

**Authors:** Abdullah, Halima Bensmail, Jessica P. Johnson, Othmane Bouhali

**Affiliations:** 1 College of Science and Engineering, Hamad Bin Khalifa University, Doha, Qatar; 2 Qatar Computing Research Institute, Hamad Bin Khalifa University, Doha, Qatar; 3 Equine Veterinary Medical Centre, Member of Qatar Foundation, Doha, Qatar

**Keywords:** equine metabolic syndrome, explainability, laminitis, machine learning, point-based risk score, risk stratification, subclinical prediction, veterinary decision support

## Abstract

Laminitis is a painful and potentially life-threatening inflammatory condition of the equine hoof, and its detection remains a major challenge in veterinary practice. Although several risk factors have been reported, the prediction of the onset of laminitis in a subclinical stage using routine clinical data is still limited. This study aims to address this gap by developing an interpretable machine learning (ML) framework for the prediction of the risk of laminitis in horses. A point-based risk scoring system was first constructed using multivariate logistic regression to quantify the contribution of clinical, physiological, and conformational variables. Subsequently, this statistical model was combined with multiple ML classifiers to improve predictive performance. Explainability techniques, including ELI5 and feature importance analysis, were applied to each model to improve transparency and support the clinical interpretation of their predictions. Among the nine classifiers evaluated, SVM achieved the highest F1-score of 0.858 ± 0.152 and MCC of 0.811 ± 0.211, with TabPFN recording the highest AUC of 0.932 ± 0.094 and Random Forest achieving the highest precision of 0.917 ± 0.180. The risk scores reached maximum values of up to 0.97, with the lameness examination right fore (LERF), hoof testers right hind (HTRH), digital pulses, rectal temperature, and age identified as the most influential predictors across all evaluated classifiers. In general, the results demonstrate that the integration of statistical risk modeling with interpretable ML enables an accurate and clinically meaningful assessment of laminitis risk. The proposed approach provides a practical decision-support tool that may help veterinarians in preventive management and improve the results of equine welfare. All the source code is available at github.

## 1 Introduction

Laminitis is recognized as a clinical syndrome that can result in a life-threatening and debilitating disease of the equine foot (1). With clinical syndromes similar to laminitis reported since 380 BC, it is now estimated to have a frequency ranging from 1.5 to 34% (2, 3). Laminitis is recognized to occur in three forms, depending on the etiology, associated with endocrinopathy, inflammatory, or mechanical causes. The etiology of laminitis is predominantly endocrinopathic in nature; it has been estimated that approximately 89% of cases arise from endocrine-related disorders, of which 58% are specifically attributed to equine metabolic syndrome (EMS), underscoring the critical need for identification of at-risk individuals (4). Genetic investigations have also been performed to identify certain heritable traits. For example, the Arabian horse has been identified as carrying a risk locus for one such endocrinopathic disease, known as EMS, therefore genetically predisposing this breed to the development of laminitis (5). Furthermore, pony breeds have been identified to have a higher risk of developing endocrinopathic laminitis compared to horse breeds (4).

Laminitis is a challenging disease to treat for many reasons, including its complex and multifactorial etiology, variable and unpredictable progression, the significant welfare concerns for the animal, as well as the risk of recurrence and prognostic uncertainty. For these reasons, the goal has now shifted toward prevention, with a focus on the identification of risk factors for the disease. Laminitis is widely accepted as a complex and multifactorial disease, and several risk factors have been identified, including obesity and endocrinopathic diseases (1). Research on the prediction of laminitis development in non-laminitic ponies has shown that basal and oral sugar-challenged serum insulin concentrations can be used to identify ponies at high risk of laminitis development (6). Similarly, horses with insulin concentrations consistent with insulin resistance have been shown to comprise two-thirds of horses presenting endocrinopathic laminitis in a first opinion/referral equine hospital (4). This same study also found that cases of endocrinopathic laminitis were significantly older than the general population of hospitals, suggesting that advancing age may also play a role (4). In addition to endocrinopathic laminitis, several other factors have been identified as associated with an increased risk of laminitis. These include recent weight gain, season, recent box-rest, previous history of laminitis (based on physical examination and radiography), foot pain after farriery, and increased time since the last anthelmintic treatment (7).

In human medicine, point-based risk scoring systems have been developed as prediabetes screening tools to identify individuals at risk of developing type 2 diabetes (8). By relying exclusively on non-invasive clinical measurements, such tools offer a simple, cost-effective, and accessible approach that can be readily implemented by both the general public and healthcare providers. Drawing on this established methodology, the present study adapts a analogous risk scoring framework for the identification of laminitis risk in horses. Non-invasive data was collected, including factors such as age, waist and hip measurements, pulse, body mass index (BMI), sex, etc (8). Logistic regression and other ML methods were used to identify significant risk factors, including age, sex, BMI, waist circumference, and blood pressure (8). The detection of prediabetes through the application of such risk scoring tools has been demonstrated to contribute significantly to reducing the overall prevalence of type 2 diabetes by enabling timely preventive interventions (8). EMS was named partly due to its resemblance to human metabolic syndrome, a group of conditions in people that include insulin resistance, obesity, and increased risk of chronic disease like Type 2 diabetes (9). The pathophysiological parallels between equine EMS and human type 2 diabetes, particularly with regard to insulin resistance, combined with the well-established association between obesity-related phenotypic indicators and basal hyperinsulinemia in horses, provide a compelling rationale for the development of an analogous risk scoring system in the equine population to facilitate the identification of individuals at risk of developing laminitis (4).

In this study, we proposed an interpretable framework for prediction of the risk of laminitis in horses by integrating statistical and ML classifiers. The risk of laminitis refers to the fact that horses may develop laminitis based on clinical features. A prediction aims to identify horses either they have laminitis or not based on features and allowing preventive care to be applied for treating the disease. A point based risk scoring system is first developed using multivariate logistic regression to quantify the contribution of clinical, physiological, and conformational factors. The statistical risk model is integrated with multiple ML classifiers to account for the non-linear relationships among clinical, physiological, and conformational features. Explainability techniques are applied to models to allow veterinarians to trace the contribution of individual predictors to the predicted laminitis risk. Model performance is assessed through cross-validation, confusion matrix analysis, and ROC curve metrics, confirming the reliability of the proposed framework as a decision-support tool in equine veterinary care. The principal contributions of this work are as follows:

- A point-based laminitis risk score is derived from multivariate logistic regression to estimate individual risk probability and identify the clinical, physiological, and conformational variables that contribute most strongly to laminitis onset.
- Multiple ML classifiers and artificial neural network models are developed for the prediction of subclinical laminitis, with each model trained and tested on the dataset to enable direct performance comparison.
- All models are evaluated using accuracy, precision, recall, MCC-score, and ROC-AUC to identify the most reliable approach for clinical application in equine veterinary practice.

## 2 Methodology

**Figure 1:**
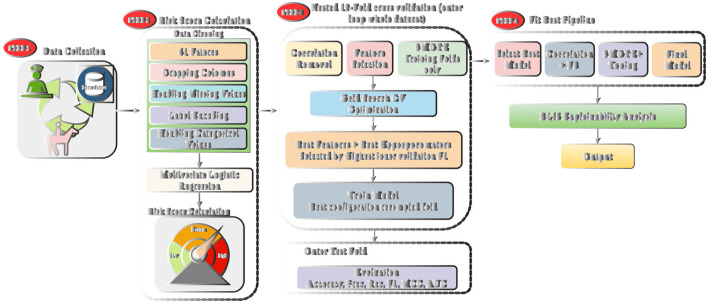
Methodology overview for laminitis risk prediction.

This study is divided into two parts. The first is a point-based risk scoring system based on a multivariate logistic regression model. This model allowed us to measure the effects of the individual variables of the clinical and physical examination and specific groups on the probability of developing laminitis. During the second step, we do classification and compare different types of supervised ML and deep learning algorithms [such as Logistic Regression (10), Support Vector Machine (SVM) (11), Random Forest (12), Gradient Boosting (13), K-Nearest Neighbors (KNN) (14), Decision Tree (15), TabPFN (16, 17), and ANN (18)] to calculate the prediction of sub-clinical laminitis (19–22). To address class imbalance in the dataset, the Synthetic Minority Oversampling Technique (SMOTE) (23) was applied to generate synthetic samples for the minority class, thereby producing a balanced training dataset and reducing the risk of model bias toward the majority class. Performance of the model measured on standard metrics such as accuracy, precision, recall, Matthews Correlation Coefficient (MCC) score, and the area under the ROC curve (AUC) (24–27). Moreover, ELI5 (explain like I'm 5) (28) was also adopted to explain the importance of features and improve the transparency of predictive models. The overall methodology is illustrated in Figure 1 and proceeds through four sequential steps. In Step 1, clinical, physiological, and conformational measurements were collected from 110 Arabian horses and structured for analysis. In Step 2, the raw dataset of 41 variables underwent preprocessing comprising column removal, label encoding, and missing value imputation before a multivariate logistic regression model was fitted on the resulting 25 features to derive a continuous individual risk score. In Step 3, a nested 10-fold cross-validation framework was applied to the full dataset. The outer loop divided the dataset into ten stratified partitions, with each test fold held out strictly for final evaluation and never exposed to any preprocessing, feature selection, or resampling operation. Within each outer training fold, an inner 10-fold cross-validation loop optimized two pipeline components simultaneously of the number of input features, searched over features using Random Forest Gini importance scores computed on the training data alone, and the model hyperparameters, tuned via grid search with the F1 score as the selection criterion (29). Pearson correlation-based feature removal and SMOTE-based oversampling were applied strictly within each inner training fold to prevent data leakage. The configuration yielding the highest inner validation F1 score was carried forward to train the final fold model, which was subsequently evaluated on the held-out outer test fold. Nine classifiers were evaluated under this framework and assessed. In Step 4, the best-performing model across all outer folds was identified based on the mean test F1-score. Within each outer fold, the model was retrained on the corresponding training split using the optimal feature subset and hyperparameters identified through inner cross-validation, and subsequently evaluated on the held-out test split. ELI5-based explainability analysis was then applied to six classifiers for which internal feature weight extraction is supported, quantifying the contribution of each selected feature to model predictions at the population level (28).

### 2.1 Study design, sample size, and study population

The control group comprised 76 clinically normal Arabian horses confirmed through physical examination, with no prior history of laminitis (adults, 3 years+, 350–450 kg). The case group comprised 34 horses with a confirmed diagnosis of laminitis based on clinical and radiographic examination. Data were collected prospectively across multiple clinical examination sessions between 2021 and 2023. To minimize the influence of diurnal variation on time-sensitive physiological measurements including heart rate, respiratory rate, and rectal temperature all examinations were conducted during routine morning veterinary rounds, with horses assessed at rest prior to any physical activity. Measurements were recorded by trained veterinary clinicians following a standardized examination protocol applied consistently across all data collection sessions. The total dataset of 110 horses is modest in size, which reflects the practical constraints of equine clinical research and confirmed laminitis cases are limited by strict diagnostic criteria and the specialized nature of the condition. Data collection was carried out on site at the Equine Veterinary Medical Center, Doha, Qatar. All procedures were approved by the Institutional Animal Care and Use Committee of the Equine Veterinary Medical Center (IACUC) (Protocol number EVMC-2019-003).

### 2.2 Data collection

Morphometric and conformational assessments included body condition score, evaluated using the Kentucky Equine Research Body Condition Score Chart, girth circumference measured posterior to the point of elbow, flank circumference measured anterior to the stifle fold, and height measured at the highest point of the withers, all recorded in centimeters. Hoof morphology was quantified through gross measurements of width, taken at the widest point across the sole from quarter to quarter, and length, taken from the toe along the sagittal midline to the base of the frog. Hoof tester response was recorded as either negative or positive. Lameness evaluation was conducted as a component of the routine clinical examination at each scheduled veterinary visit, prior to and independent of any formal laminitis diagnosis. Each horse was assessed at walk and trot on a firm surface, and lameness severity was graded according to the American Association of Equine Practitioners (AAEP) Lameness Scale (0–5) (30). The AAEP Lameness Scale spans six grades: Grade 0 (no perceptible lameness) through Grade 5 (complete inability to bear weight). In this study, control horses predominantly scored 0, while laminitis cases presented with scores between 3 and 4 lameness consistently observable at trot and detectable at walk reflecting to moderate clinical severity captured by the dataset (30). Lameness scores were recorded as a routine clinical observation rather than as a diagnostic criterion for laminitis; in the control group, the predominant score was 0, consistent with their clinically normal status. The inclusion of lameness examination scores as predictors was intended to capture subtle gait abnormalities that may antedate a formal laminitis diagnosis, rather than to classify horses with overt clinical disease.

### 2.3 Data organization

The recorded variables were organized into five clinical categories as follows:

**Demographic characteristics:**

- Age (years): < 5 very young; 5–9 young; 10–14 middle-aged; 15–20 old; >20 geriatric.
- Gender: stallion; gelding; mare.

**Vital signs:**

- Heart rate (bpm): < 24 low; 24–44 normal; >44 high.
- Respiratory rate (bpm): < 8 low; 8–16 normal; >16 high.
- Rectal temperature (°C): < 37.5 low; 37.5–38.5 normal; >38.5 high.

**Physical examination findings:**

- Gut sounds: decreased; normal; increased.
- Digital pulses: normal; increased.
- Hoof tester response: negative; positive; not evaluated.

**Body measurements:**

- Body weight (kg): < 350 low; 350–450 normal; >450 high.
- Body condition score: < 5 thin; 5–7 moderate; 8–9 fat.
- Hoof length (cm): < 12 short; 12–14 normal; >14 long.
- Hoof width (cm): < 11 narrow; 11–12 normal; >12 wide.

**Lameness assessment:**

- Lameness examination score: 0 not lame; 3 lame on trot; 4 lame on walk.

### 2.4 Data analysis

The dataset comprised 110 horses in total in which 76 clinically normal controls and 34 confirmed laminitis cases. The data passed through four preprocessing steps before model training. First, rows missing more than 90% of their values were removed. Administrative and non-clinical columns horse identification numbers, study dates, and photograph fields were then dropped, reducing the feature set from 41 to 25 clinically relevant variables. Categorical variables, including sex, gut sounds, digital pulses, and hoof tester responses, were encoded numerically using label encoding and predefined ordinal mappings, with remaining missing values filled by column mode or median. The control and laminitis groups were then concatenated and shuffled into a single dataset.

### 2.5 Statistical analysis for risk score calculation

To measure the point system and the calculated risk scores, a multivariate logistic regression was used on 25 features that were selected and extracted in section 2.4 by data analysis (8). It was created with the help of a multivariate logistic regression model in which these 25 features which is age, sex, respiratory rate, rectal temperature, gut sounds, digital pulses, body weight, body condition scoring, length right fore foot (Length RF), length left fore foot (Length LF), length right hind foot (Length RH), length left hind foot (Length LH), width right fore foot (Width RF), width left fore foot (Width LF), width right hind foot (Width RH), width left hind foot (Width LH), hoof testers right fore (HTRF), hoof testers left fore (HTLF), HTRH, hoof testers left hind (HTLH), LERF, lameness examination left fore (LELF), lameness examination right hind (LERH), and lameness examination left hind (LELH) were considered critical risk factors. Following the preprocessing pipeline described in Section 2.4, the final dataset comprised 110 horses in which 76 clinically normal controls and 34 confirmed laminitis cases. The logistic regression risk scoring procedure is described in the sections that follow (31, 32).

#### 2.5.1 Estimate the parameters of the multivariate logistic regression model

Multivariate logistic regression was used to estimate two sets of parameters: the regression coefficients β and the intercept β_0_, derived from the clinical, physiological, and conformational features described in Section 2.4 (33). The sigmoid function converts the linear combination of these predictors into a probability between 0 and 1 (34). The predicted probability of having laminitis *p* is given by Equation 1.


$$\begin{array}{l} p=\frac{1}{1+exp\left(-\left(\beta_{0}+\beta_{1}x_{1}+\beta_{2}x_{2}+\beta_{3}x_{3}\cdots +\beta_{p}x_{p}\right)\right)} \end{array}$$ (1)

where, β_0_ is the intercept and β_1_, β_2_, β_3_, ⋯β_*p*_ are the coefficients for the characteristics of the predictor variables *x*_1_, *x*_2_, *x*_3_, …, *x*_*p*_.

#### 2.5.2 Classify the risk factors and obtain reference values for each category

Each risk factor was categorized into clinically meaningful groups, and a representative midpoint value was assigned to each category for numerical analysis (8). For scoring purposes, the lowest-risk category of each factor was defined as the reference baseline and assigned a score of zero. The selected baseline categories for all risk factors are summarized in Table 1.

**Table 1:** Logistic regression analysis of risk factors associated with laminitis.

Risk factor	Categories	Reference value (*W*_*ij*_)	*p*-value	β_*i*_	β_*i*_(*Wij*−*W*_*iREF*_)	*Points*_*ij*_=β_*i*_(*Wij*−*W*_*iREF*_)/*B*	Risk score
Age (years)	0–5	2.5 = *W*_1*REF*_	0.9323	0.032332	0	0	0.50
	6–11	8.5			0.193992	1	0.54
	12–17	14.5			0.387984	2	0.58
	18–23	20.5			0.581976	4	0.65
	23–28	25.5			0.743636	5	**0.69**
Sex	0	0 = *W*_2*REF*_	0.9916	0.018435	0	0	0.50
	1	1			0.018435	0	0.50
	2	2			0.03687	0	0.50
	3	3			0.055305	0	0.50
Heart rate	20–29	25.0 = *W*_2*REF*_	0.9441	0.035535	0	0	0.50
	30–44	37.0			0.426420	3	0.61
	44–60	52.0			0.959445	6	0.72
	61–88	75.0			1.776750	11	**0.85**
Respiratory rate	8–16	12.0 = *W*_3*REF*_	0.8799	0.058176	0	0	0.50
	17–30	24.0			0.698112	4	0.65
	31–50	40.0			1.628928	10	**0.83**
	51–76	63.0			2.966976	18	**0.95**
Rectal temperature	35.8–36.9	36.4 = *W*_4*REF*_	0.9661	–0.185257	0	0	0.50
	37.0–38.0	37.5			–0.203783	–1	0.46
	38.1–38.5	38.3			–0.351988	–2	0.42
Gut sounds	0	0 = *W*_5*REF*_	0.9935	–0.066299	0	0	0.50
	1	1			–0.066299	0	0.50
Digital pulses	0	0.0 = *W*_6*REF*_	0.9671	0.186168	0	0	0.50
	1	1.0			0.186168	–4	**0.54**
Body weight	245–300	272.0 = *W*_7*REF*_	0.8792	–0.005099	0	0	0.50
	301–400	350.0			–0.397722	–2	0.42
	401–500	450.0			–0.907622	–6	0.27
	501–567	534.0			–1.335938	–8	0.21
Body condition scoring	1–3	2.0 = *W*_8*REF*_	0.9063	0.141494	0	0	0.50
	4–6	5.0			0.424482	3	0.61
	7–9	8.0			0.848964	5	**0.69**
LengthRF	0–5	2.5 = *W*_9*REF*_	0.9896	–0.059366	0	0	0.50
	5.1–10	7.5			0.296830	–2	0.42
	10.1–14.5	12.3			–0.581786	–4	0.34
LengthLF	0–5	2.5 = *W*_9*REF*_	0.9862	–0.096161	0	0	0.50
	5.1–10	7.5			–0.480805	–3	0.38
	10.1–14.5	12.3			–0.942377	–6	0.27
LengthRH	0–5	2.5 = *W*_9*REF*_	0.9985	–0.000161	0	0	0.50
	5.1–10	7.5			–0.000805	0	0.50
	10.1–14.5	12.3			–0.001578	5	0.50
LengthLH	0–5	2.5 = *W*_9*REF*_	0.9966	0.017474	0	0	0.50
	5.1–10	7.5			0.08737	1	0.54
	10.1–14.5	12.3			0.171245	1	0.54
Width RF	0–5	2.5 = *W*_14*REF*_	0.9646	–0.025841	0	0	0.50
	5.1–10	7.5			–0.129205	–1	0.46
	10.1–14.5	12.0			–0.2454895	–2	0.42
Width LF	0–5	2.5 = *W*_14*REF*_	0.9961	0.020859	0	0	0.50
	5.1–10	7.5			0.104295	1	0.54
	10.1–14.5	12.0			0.198161	1	0.54
Width RH	0–5	2.5 = *W*_14*REF*_	0.9884	0.026967	0	0	0.50
	5.1–10	7.5			0.134835	1	0.54
	10.1–14.5	12.0			0.256187	2	0.58
Width LH	0–5	2.5 = *W*_14*REF*_	0.9968	0.008199	0	0	0.50
	5.1–10	7.5			0.040995	1	0.50
	10.1–14.5	12.0			0.077891	0	0.50
HTRF	0	0.0 = *W*_9*REF*_	0.9852	–0.123157	0	0	0.50
	1	1.0			–0.123157	–1	0.46
	2	2.0			–0.246314	–2	0.42
HTLF	0	0.0 = *W*_9*REF*_	0.9897	–0.086529	0	0	0.50
	1	1.0			–0.086529	–1	0.46
	2	2.0			–0.173058	–1	0.46
	3	3.0			–0.259587	–2	0.42
HTRH	0	0.0 = *W*_9*REF*_	0.9452	–0.360465	0	0	0.40
	1	1.0			–0.360465	–2	0.42
HTLH	0	0.0 = *W*_9*REF*_	0.9471	–0.352151	0	0	0.50
	1	1.0			–0.352151	–2	0.42
LERF =	0	0.0 = *W*_9*REF*_	0.6614	0.744567	0	0	0.50
	3	3.0			2.233701	14	**0.90**
	4	4.0			2.978268	18	**0.95**
LELF	0	0.0 = *W*_9*REF*_	0.6307	0.872817	0	0	0.50
	3	3.0			2.618451	16	**0.93**
	4	4.0			3.491268	22	**0.97**
LERH	0	0.0 = *W*_9*REF*_	0.6340	0.781217	0	0	0.50
	3	3.0			2.343651	14	**0.90**
	4	4.0			3.124868	19	**0.95**
LELH	0	0.0 = *W*_9*REF*_	0.6792	0.818301	0	0	0.50
	3	3.0			2.454903	15	**0.92**
	4	4.0			3.273204	20	**0.96**

Bold values indicate the best performing results.

#### 2.5.3 Find the distance of each category from the base category in regression units

For each risk factor *i*, the distance of category *j* from the baseline was computed as β_*i*_(*W*_*ij*_−*W*_*iREF*_), where *W*_*ij*_ is the value of category *j* of risk factor *i*. This quantity reflects how much each category shifts the predicted laminitis probability relative to the lowest-risk baseline.

#### 2.5.4 Set the constant, *B*

The constant *B* converts regression units into points on the risk score scale. One point was defined as the change in risk associated with a 5-year increase in age, a clinically interpretable unit of risk progression in horses.


$$\begin{array}{l} B=5*\left(0.032332\right)=0.16166 \end{array}$$ (2)

#### 2.5.5 Determine points associated with each of the categories of risk factors

Points were assigned to each category *j* of risk factor *i* as.


$$\begin{array}{l} Points_{ij}=\beta_{i}\left(W_{ij}-W_{iREF}\right)/B \end{array}$$ (3)

Values were rounded to the nearest integer. The full point assignments are presented in Table 1.

#### 2.5.6 Determine points associated with each risk factor category

The probability related to each point total is now determined. We first have to calculate the theoretical range of the number of points with reference to the calculated point system in Section 2.5.5. The possible minimum and maximum point totals in this system are –9 and 23 respectively. Now we should add a risk estimate to each total point with the help of the multiple logistic regression Equation 1.

The total point score *S* is a scaled representation of the linear predictor from the logistic regression model. Specifically, the score can be expressed as:


$$\begin{array}{l} S=\frac{logit\left(p\right)-\beta_{0}}{B} \end{array}$$ (4)

where β_0_ is the intercept and *B* is a scaling constant. Thus, the score is obtained by subtracting the intercept from the logit of the predicted probability of laminitis and rescaling the result by $\frac{1}{B}$. The predicted probability can then be recovered by substituting |*beta*_0_+*B*×*S* into Equation 4 (32).

### 2.6 Classification method

The ML classification pipeline was implemented in Python (Python Programming Language, Wilmington, Delaware, United States) and evaluated across nine classifiers: Logistic Regression, SVM, KNN, Random Forest, Decision Tree, Gradient Boosting, XGBoost, ANN, and TabPFN. To prevent data leakage and ensure that performance estimates reflect genuine generalization, a nested 10-fold cross-validation strategy was applied throughout.

#### 2.6.1 Nested cross-validation

The outer loop divided the full dataset into five stratified folds, preserving the proportion of laminitis cases and controls across all partitions. In each outer iteration, nine folds served as the training set and one was held out strictly for testing. The test fold was never used to inform any preprocessing, feature selection, or resampling decision. Within each outer training fold, an inner 10-fold cross-validation loop optimized two pipeline components simultaneously: the number of input features and the model hyperparameters. The inner loop searched over candidate feature counts of 10, 12, 15, 18, and 20. For each candidate count, the top-ranked features were selected by Random Forest Gini impurity scores computed on the inner training data alone, and a grid search over model-specific hyperparameters was conducted using the inner validation folds. The F1 score served as the optimization criterion and a metric that balances precision and recall and is better suited than accuracy when class sizes differ substantially, as in this dataset (34 laminitis cases, 76 controls). The feature count and hyperparameter combination yielding the highest mean inner validation F1 score was carried forward to the outer test fold (35, 36).

#### 2.6.2 Preprocessing within each training fold

All preprocessing steps were confined to the outer training fold in each iteration. First, a Pearson correlation matrix was computed on the training data, and feature pairs with a correlation coefficient above 0.7 were flagged. The less clinically informative variable from each pair was removed, reducing the initial set of 25 variables. Second, Random Forest Gini impurity scores were computed on the remaining features, and the top *n* variables where *n* was determined by the inner loop were retained for model training. Third, SMOTE was applied to the training fold to address class imbalance, generating synthetic minority class samples through k-nearest neighbor interpolation. SMOTE was applied after feature selection and exclusively to the training data, so no synthetic samples derived from the test fold entered the training process (37, 38).

#### 2.6.3 Model training and evaluation

Each classifier was trained on the resampled training fold using the hyperparameters selected in the inner loop and evaluated on the held-out test fold. Six metrics were recorded for each fold: accuracy, precision, recall, F1-score, MCC, and ROC-AUC. Training and validation metrics were also recorded to monitor potential overfitting. Although model selection was based on the mean test F1-score, MCC was additionally reported because it accounts for all four cells of the confusion matrix and remains reliable under class imbalance, where accuracy and F1-score can be misleading (26). After nested cross-validation, the model with the highest mean test F1-score across the ten outer folds was selected as the best-performing pipeline. This pipeline was subsequently refitted on the full dataset to produce a deployable model and to enable analysis of the selected features and coefficients. Specifically, correlation-based feature removal and Random Forest–based feature selection were applied to the full dataset, followed by SMOTE to address class imbalance. Hyperparameter tuning was then performed using cross-validation on the full dataset, and the final model was trained using the resulting optimal configuration. The outer test folds were used exclusively for performance evaluation during nested cross-validation and were not involved in the final model fitting.

#### 2.6.4 Explainability analysis

ELI5 was applied to six classifiers that support extraction of internal feature weights, namely Random Forest, Logistic Regression, SVM, Decision Tree, Gradient Boosting, and XGBoost. For tree-based models, ELI5 provides feature importance based on Gini impurity, while for linear models it extracts signed coefficients. These measures quantify the contribution of each selected feature to model predictions at the population level. TabPFN was excluded from this analysis, as it does not expose feature weights in a format compatible with ELI5. All feature importance values were computed by fitting each classifier on the full dataset using the final selected features. SMOTE was not applied at this stage; instead, class imbalance was handled using the class_weight = “balanced~ parameter to avoid introducing synthetic samples into the explainability analysis (28, 39).

#### 2.6.5 Evaluation metrics

To evaluate the performance of the classification algorithms, we used some tools, which are accuracy, precision, recall, MCC score, and ROC curve (26, 27, 40, 41). These metrics are defined below.

- **Accuracy:** accuracy is the proportion of the correct prediction of the model over the total number of predictions made.

$$Accuracy=\frac{TP+TN}{TP+TN+FP+FN}$$

- **Precision:** is the proportion of the true positive prediction by the model over all positive prediction. This is basically the accuracy of the positive prediction.

$$Precision=\frac{TP}{TP+FP}$$

- **Recall:** is the metric that measures the correct prediction of the model from all actual positive samples in the dataset.

$$Recall=\frac{TP}{TP+FN}$$

- **MCC:** performance metric that measures the quality of a classification model by considering all elements of the confusion matrix (TP, TN, FP, FN).

$$MCC=\frac{TP\times TN-FP\times FN}{\sqrt{\left(TP+FP\right)\left(TP+FN\right)\left(TN+FP\right)\left(TN+FN\right)}}$$

- **Confusion matrix:** is used to evaluate the quality and accuracy of classification models. It provides us with the result of the forecasts of a model against the actual results.
- **ROC curve:** a Receiver Operating Characteristic (ROC) curve is a ML chart that demonstrates how a binary classifier works at various discrimination levels. It graphs the True Positive Rate (TPR) (sensitivity) versus the False Positive Rate (FPR) (1 minus specificity) with different threshold settings to aid in viewing the trade-off between the percentage number of cases identified correctly as positive and the percentage number of false positives.

### 2.7 Computational setup

All analyses were conducted on a MacBook Pro (2.4 GHz 8-Core Intel Core i9 processor, 32 GB 2400 MHz DDR4 memory) using Python. The classifiers evaluated included Logistic Regression (10), Random Forest (12), SVM (11), Gradient Boosting (13), XGBoost (42), KNN (14), Decision Tree (15), TabPFN (16, 17), and ANN (43, 44).

## 3 Results and discussion

This section presents the results of the risk assessment conducted using multivariate logistic regression and examines the classification of laminitis through various ML and deep learning models. Furthermore, a comprehensive comparison of the performance of all classification algorithms is provided and discussed in detail in the following.

### 3.1 Multivariate logistic regression to evaluate risk score

Table 1 presents the results of the multivariate logistic regression analysis used to identify the clinical and physiological variables most strongly associated with laminitis risk. Each feature was modeled relative to a reference category, yielding a regression coefficient β, weighted difference, point score, and computed risk score. Age, heart rate, respiratory rate, body condition score, and lameness examination were the strongest predictors. All five carried positive regression coefficients, meaning that higher values or more severe categories corresponded to greater predicted risk. Horses aged 21–25 years reached a risk score of 0.69, consistent with the metabolic and structural hoof changes associated with aging. Heart rate between 61–88 bpm and respiratory rate between 51–76 bpm produced the highest positive coefficients in the model, with corresponding risk scores of 0.85 and 0.93 values that reflect the physiological stress patterns seen in systemic inflammation. Body condition scores of 7–9 were similarly linked to elevated risk (score 0.69), consistent with the established relationship between obesity and endocrinopathic laminitis. Rectal temperature and intestinal sounds carried negative β values within their normal physiological ranges, suggesting a lower or protective association at those levels. Lameness examination variables LERF, LELF, LERH, and LELH provided localized diagnostic information, with overt lameness signs associated with risk scores reaching 0.97.

The logistic regression model quantified laminitis risk from routine clinical measurements and produced interpretable risk scores that allow veterinarians to stratify horses by their probability of developing the condition. However, logistic regression assumes linear relationships between predictors and the outcome, which may not fully capture the interactions among multiple clinical variables. To address this, nine ML and ANN classifiers were subsequently applied to the same dataset. Each model was trained on the resampled training set and evaluated on the held out test set using accuracy, MCC score, precision, recall, and ROC-AUC.

The logistic regression risk scoring system and the ML classification pipeline operate as two independent components of the proposed framework. The first component quantifies individual laminitis risk from all 25 clinical features and produces an interpretable point-based score, while the ML classifiers are trained independently on the features selected through the nested cross-validation pipeline to provide automated and accurate classification. The following section presents a detailed performance comparison of all nine classifiers. The following section presents a detailed performance comparison of all nine classifiers, together with ELI5-based explainability analysis to identify which features drove each model's predictions. Although none of the predictors reached conventional statistical significance (*p* < 0.05), this is attributable to the limited statistical power of the small sample size (*n* = 110) rather than the absence of clinically meaningful effects. The regression coefficients and derived risk scores remain interpretable as measures of relative risk contribution, and the p-values are reported in Table 1 for transparency.

### 3.2 Performance comparison of ML models

Table 2 reports the average 10-fold nested cross-validation test performance of all nine classifiers after excluding the four lameness examination scores from the feature set due to the part of diagnosis. Several classifiers retained meaningful predictive ability from the remaining physiological and conformational measurements alone. SVM achieved the strongest overall performance, recording an F1-score of 0.858 ± 0.152, an MCC of 0.811 ± 0.211, and an AUC of 0.930 ± 0.086 the lowest standard deviation among all models, indicating consistent and stable generalization across folds. Random Forest performed comparably, achieving an F1-score of 0.843 ± 0.210 and the highest AUC of 0.932 ± 0.105, confirming its ability to identify informative feature combinations from the remaining clinical variables. TabPFN followed closely with an F1-score of 0.816 ± 0.214 and an AUC of 0.932 ± 0.094, jointly matching Random Forest on AUC.

**Table 2:** Average 10-fold cross-validation test performance of all models analysis.

Model	Sel. features	Accuracy	Precision	Recall	F1 score	MCC	AUC
ANN	12	0.8453 ± 0.1217	0.7634 ± 0.2375	0.7251 ± 0.2224	0.7355 ± 0.2129	0.6347 ± 0.2979	0.8751 ± 0.1629
Decision Tree	12	0.8726 ± 0.1436	0.8200 ± 0.2627	0.8167 ± 0.2250	0.8017 ± 0.2137	0.7241 ± 0.3177	0.8628 ± 0.1431
Gradient Boosting	12	0.8817 ± 0.1489	0.8417 ± 0.2467	0.7917 ± 0.2461	0.8064 ± 0.2314	0.7304 ± 0.3376	0.8685 ± 0.1727
KNN	11	0.8544 ± 0.1370	0.7542 ± 0.2101	**0.8833 ± 0.2195**	0.7923 ± 0.1885	0.7153 ± 0.2475	0.9088 ± 0.1670
Logistic Regression	13	0.8453 ± 0.1489	0.7484 ± 0.2585	0.7751 ± 0.2224	0.7547 ± 0.2302	0.6478 ± 0.3369	0.8868 ± 0.1618
Random Forest	12	0.9181 ± 0.1089	**0.9167 ± 0.1800**	0.7917 ± 0.2461	0.8428 ± 0.2101	0.7977 ± 0.2760	0.9322 ± 0.1048
SVM	11	**0.9181 ± 0.0905**	0.8967 ± 0.1808	0.8418 ± 0.1686	**0.8584 ± 0.1517**	**0.8106 ± 0.2107**	0.9297 ± 0.0863
TabPFN	20	0.8908 ± 0.1343	0.8467 ± 0.2451	0.8084 ± 0.2292	0.8156 ± 0.2144	0.7491 ± 0.3104	**0.9315 ± 0.0942**
XGBoost	11	0.8727 ± 0.1670	0.8367 ± 0.2602	0.7917 ± 0.2461	0.7992 ± 0.2321	0.7207 ± 0.3545	0.9006 ± 0.1371

Lameness examination scores (LERF, LELF, LERH, LELH) excluded from all feature sets.

Values reported as mean ± standard deviation across 10 outer folds.

“Sel. Features" represents the most frequently selected feature count across the 10 outer folds. Bold values indicate the best performing results.

Gradient Boosting and Decision Tree produced moderate F1-scores of 0.806 ± 0.231 and 0.802 ± 0.214 respectively, with high standard deviations reflecting sensitivity to fold composition on small datasets. KNN achieved the highest recall of 0.883 ± 0.220 but at the cost of lower precision and high fold-to-fold variance. XGBoost recorded an F1-score of 0.799 ± 0.232 and an AUC of 0.901 ± 0.137, showing a more pronounced reduction in performance compared to SVM and Random Forest. Logistic Regression and ANN were the weakest performers, recording F1-scores of 0.755 ± 0.230 and 0.736 ± 0.213 respectively and the lowest MCC values in the comparison. SVM is identified as the best performing model in this analysis on the basis of its highest F1-score, highest MCC, and lowest fold-to-fold variance confirming that the framework retains clinically meaningful discriminative ability from hoof tester responses, digital pulses, rectal temperature, and conformational measurements alone. The fact that logistic regression performs much worse than the SVM, indicates the importance of non-linear relationships between inputs and the probability of having laminitis.

#### 3.2.1 Class wise performance analysis based on confusion matrices

**Figure 2:**
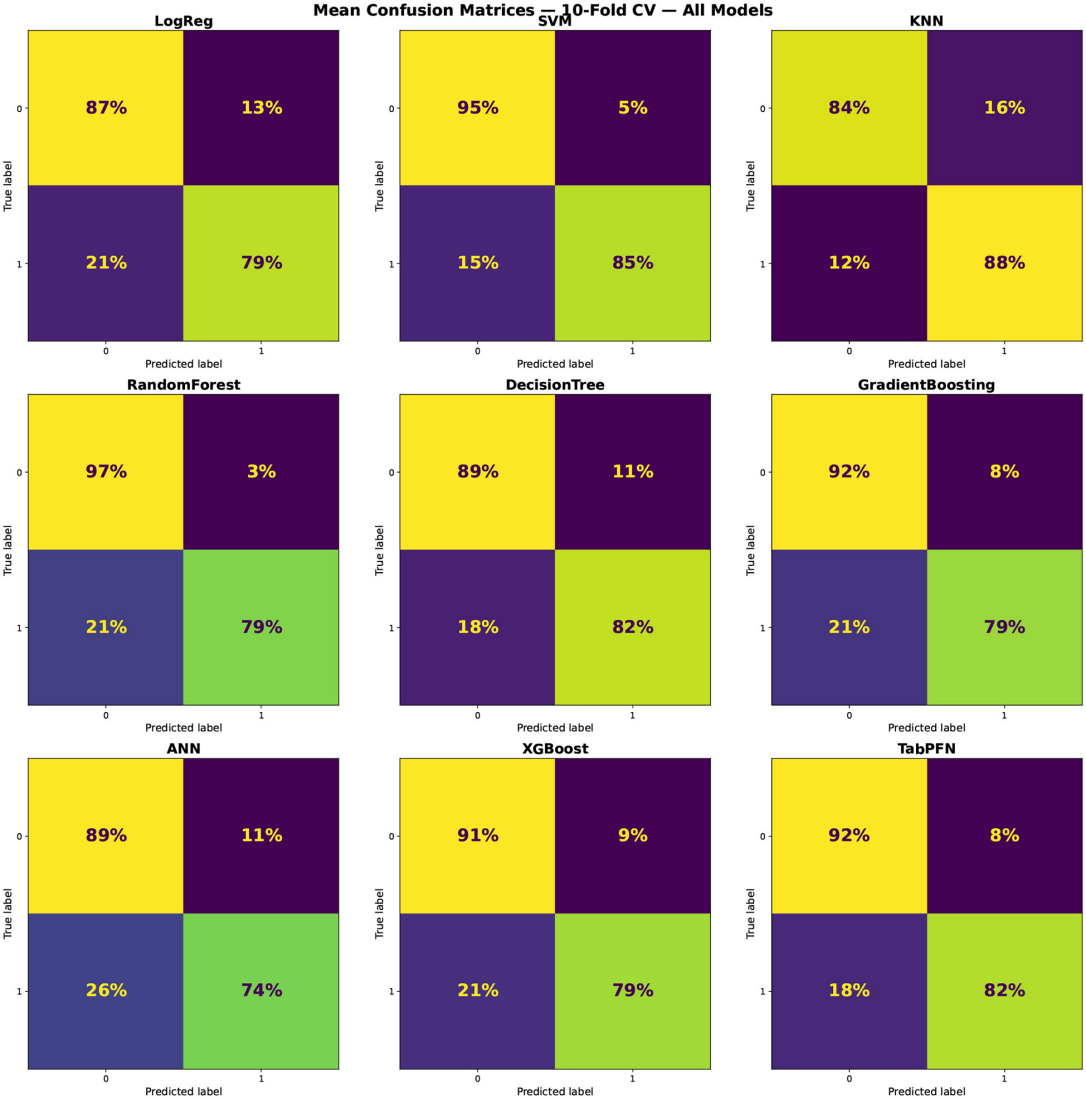
Mean confusion matrices averaged across five cross-validation folds for all nine classifiers, showing the percentage of correctly and incorrectly classified laminitis cases (class 1) and control horses (class 0).

Figure 2 presents the mean confusion matrices averaged across ten nested cross-validation folds for all nine classifiers. The most immediate observation across all models is a substantial increase in false-negative rates compared to the primary analysis meaning a greater proportion of laminitis cases are misclassified as healthy when lameness information is unavailable. In a clinical screening context, this distinction matters a false negative represents a horse with active laminitis that leaves a veterinary examination without intervention, with direct consequences for disease progression and animal welfare.

SVM produced the most balanced class-wise performance in this analysis. Rather than sacrificing sensitivity for specificity or vice versa, a trade-off that several other models made explicitly SVM maintained reasonable identification of laminitis cases while keeping the false-alarm rate among the lowest in the comparison. This balance is what makes it the most practically deployable classifier when lameness scores are not recorded, as it avoids both the systematic under-detection of affected horses and the unnecessary treatment of healthy ones. Random Forest achieved the highest specificity of all nine models, correctly identifying nearly all control horses. This came at the cost of a higher false-negative rate, reflecting a well-documented tendency of ensemble methods to favor the majority class when the most discriminative features are removed. The pattern is clinically instructive high specificity in a screening tool is less valuable than it appears if it is achieved by under-calling the very cases the tool is designed to detect.

KNN showed the reverse profile the highest sensitivity but the lowest specificity in the comparison. It correctly identified the greatest proportion of laminitis cases but misclassified a notable share of healthy horses as positive. While prioritizing sensitivity is defensible in a clinical screening context, a high false-positive rate carries its own costs in veterinary practice unnecessary diagnostic workup, treatment, and owner concern that reduce the practical utility of the classifier. ANN is the weakest performer, producing the highest false-negative rate of any model. Its failure to reliably identify laminitis cases without lameness input is consistent with the known vulnerability of neural networks to overfitting on small datasets, where the number of parameters substantially exceeds the effective training sample size after stratified partitioning.

The confusion matrices collectively show that the framework retains genuine discriminative ability without lameness scores SVM, in particular, correctly identifies the majority of laminitis cases from physiological and conformational features alone. At the same time, the consistent increase in false-negative rates across all models reinforces the clinical importance of lameness examination as part of any routine screening protocol. Hoof tester response, digital pulses, and rectal temperature provide meaningful independent signal, but they do not fully substitute for the direct assessment of gait and weight-bearing asymmetry that lameness grading provides.

#### 3.2.2 Comparative ROC analysis

**Figure 3:**
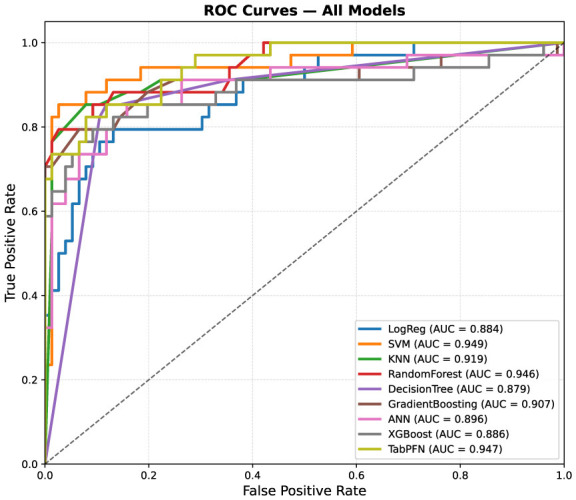
Receiver oerating characteristic (ROC) curves for test sets.

Figure 3 presents the ROC curves for all nine classifiers evaluated across ten cross-validation folds. At low false positive rates (FPR < 0.2), SVM and TabPFN demonstrate the steepest increases in true positive rate, indicating superior sensitivity in the clinically relevant region where false positives must be minimized. These models achieved the highest AUC values of 0.949 and 0.947, respectively, followed closely by Random Forest of AUC = 0.946, suggesting that both kernel-based and ensemble methods effectively capture non-linear relationships in the data.

In contrast, KNN of AUC 0.919 and Gradient Boosting of AUC 0.907 exhibit more gradual increases in true positive rate, reflecting performance distributed more evenly across decision thresholds. ANN of AUC 0.896, XGBoost of AUC 0.886, and Logistic Regression of AUC 0.884 show relatively flatter curves in the low-FPR region, indicating reduced sensitivity under conservative operating conditions. Decision Tree achieved the lowest AUC of 0.879, with a step-like curve characteristic of its discrete decision boundaries and limited probability calibration. Overall, the ROC analysis highlights the advantage of non-linear models, particularly SVM and TabPFN, in achieving strong discrimination at low FPR, which is critical for reliable clinical screening applications.

### 3.3 Model interpretability

Table 3 presents the ELI5 feature importance weights extracted from six classifiers fitted on the full dataset after excluding lameness examination scores. The consistency of feature rankings across structurally distinct model architectures strengthens confidence in the clinical relevance of the identified predictors. The Hoof tester response of the right hind foot (HTRH) emerged as the dominant predictor across all six classifiers, receiving the highest importance weight in all tree-based models and the largest negative coefficient in Logistic Regression (−2.349) and SVM (−1.616). This is clinically coherent, as the absence of pain on digital compression is associated with reduced laminitis risk, while a positive response is widely recognized as an indicator of active lamellar inflammation.

**Table 3:** ELI5 feature importance weights across six classifiers fitted on the full dataset (lameness examination scores excluded).

Feature	Random Forest	Logistic Regression	SVM	Decision Tree	Gradient Boosting	XGBoost
HTRH	0.321	−2.349	−1.616	0.563	0.583	0.686
Digitalpulses	0.179	1.985	0.886	0.114	0.134	0.112
Rectal temperature	0.123	0.843	0.525	0.083	0.064	0.013
Bodyweight (kg)	0.070	−0.005	−0.006	0.069	0.084	0.011
Age (years)	0.068	0.049	0.005	0.023	0.026	0.009
WidthRF	0.055	−0.280	−0.193	0.070	0.027	0.011
Respiratoryrate	0.054	0.074	0.045	0.000	0.003	0.003
WidthRH	0.051	0.400	0.236	0.000	0.019	0.012
HTRF	0.043	−0.382	−0.163	0.056	0.059	0.131
HeartRate	0.036	+0.004	+0.013	0.023	0.001	0.011

Lameness examination scores (LERF, LELF, LERH, LELH) excluded from this analysis.

Positive coefficients (Logistic Regression, SVM) indicate association with laminitis risk.

Negative coefficients indicate association with lower predicted risk.

TabPFN excluded—does not expose internal feature weights compatible with ELI5.

Digital pulses ranked second across five of the six classifiers, consistent with their established role as a clinical indicator of lamellar inflammation. Rectal temperature was consistently ranked among the top predictors, reflecting the systemic inflammatory component of laminitis. Bodyweight and age contributed more modestly, in line with previously reported risk factors associated with laminitis development (4, 5). In contrast, heart rate was consistently the lowest-ranked feature across all models, likely reflecting its high physiological variability and sensitivity to external factors such as handling stress.

## 4 Conclusion

This study developed a two-component framework for identifying laminitis risk in horses from routine non-invasive clinical, physiological, and conformational measurements collected from 110 Arabian horses. The first component constructed a point-based risk scoring system using multivariate logistic regression, with individual risk scores reaching up to 0.97. Age, heart rate, respiratory rate, body condition score, and lameness examination measures were identified as the most influential contributors, providing veterinarians with a transparent, equipment-free screening tool applicable during routine examination without computational infrastructure.

The second component evaluated nine ML classifiers under a nested 10-fold cross-validation framework after excluding lameness examination scores, assessing the framework's ability to identify laminitis risk from physiological and conformational measurements alone. SVM emerged as the best performing model, achieving the highest F1-score and MCC with the lowest standard deviation among all models confirming stable and consistent generalization across folds. TabPFN achieved the highest AUC, while Random Forest recorded the highest precision. ELI5 explainability analysis identified hoof testers right hind as the dominant predictor across all six classifiers for which weight extraction was supported, followed by digital pulses and rectal temperature confirming that the framework captures genuine biological signal from routine non-invasive measurements that can be recorded by any veterinarian during a standard clinical visit. The two components together provide veterinarians with a practical, data-driven decision-support tool for laminitis risk identification in routine clinical practice. The cross-sectional single-center design and modest sample size are acknowledged limitations, and prospective validation on a larger, multi-center, multi-breed dataset remains the necessary next step before broader clinical deployment.

## Data Availability

The datasets presented in this article are not readily available because dataset is private. Requests to access the datasets should be directed to Jessica P. Johnson.
