# Supplementary material for: A point-based laminitis risk scoring system and machine learning framework for prediction of laminitis in horses
Source: Front Vet Sci. 2026 Jul 1;13:1809041. doi: 10.3389/fvets.2026.1809041 (PMC13368518; doi:10.3389/fvets.2026.1809041)
Supplement: Supplementary file 1 [file Table_1.docx]

**Supplementary Data**

Table 1 shows the distribution of laminitis prevalence across risk in both the training and test sets. In the training set, prevalence increased monotonically with assigned risk level 39.47\% in the low-risk group, 46.34\% in the medium-risk group, and 64.86\% in the high-risk group. This gradient confirms that the logistic regression scoring system stratifies horses in a clinically meaningful way horses assigned to higher risk categories were, in practice, substantially more likely to develop laminitis. The test set preserved the general direction of this relationship, with higher risk categories associated with greater laminitis prevalence. However, the specific prevalence rates did not follow the same monotonic pattern observed in training (0.00\%, 30.00\%, and 16.67\% for low, medium, and high risk groups respectively). The inversion between the medium and high-risk groups in the test set is attributed to the small number of horses in each stratum after the 80/20 split minor shifts in a handful of cases produce large percentage changes when group sizes are small. This does not indicate model failure; rather, it reflects the statistical instability inherent in evaluating stratified prevalence on a limited test sample. Validation on a larger, independent dataset is needed to confirm the reliability of the risk stratification across all three categories.

Table 1Laminitis prevalence in Training and Testing populations based on risk levels

| Dataset | Risk Level | Control | Case | Total | % of Laminitis |
| --- | --- | --- | --- | --- | --- |
| Training | Low Risk (<$11$) | 23 | 15 | 38 | 39.47% |
|  | Moderate Risk ($11-19$) | 22 | 19 | 41 | 46.34% |
|  | High Risk ($\geq19$) | 13 | 24 | 37 | 64.86% |
| Testing | Low Risk (<$11$) | 6 | 0 | 6 | 00% |
|  | Moderate Risk ($11-19$) | 7 | 3 | 10 | 30.00% |
|  | High Risk ($\geq19$) | 5 | 1 | 6 | 16.67% |
